# Major Depression in Chinese Medicine Outpatients with Stagnation Syndrome: Prevalence and the Impairments in Well-Being

**DOI:** 10.1155/2018/7234101

**Published:** 2018-09-13

**Authors:** Siu-Man Ng, Ling-Li Leng

**Affiliations:** The University of Hong Kong, Hong Kong

## Abstract

Stagnation syndrome, a diagnostic entity in traditional Chinese medicine (TCM), has been long regarded as the TCM counterpart of major depression in Western medicine. The study investigated the prevalence of major depression among stagnation syndrome patients and evaluated their well-being and functioning outcomes. In total, 117 patients diagnosed with stagnation syndrome were measured using Stagnation Scale, the Patient Health Questionnaire-9 (PHQ-9), and the Body-Mind-Spirit Well-Being Inventory. Results indicate major depression among stagnation syndrome patients was characterized by a high co-occurrence rate and worse physical, mental, and functional outcomes. More than one-quarter (26.5%) of the patients met the DSM-V diagnostic criteria for major depression and over half (53%) exceeded the PHQ-9 cutoff (score above 10) for moderate/severe depression symptoms. The wellness of the stagnation syndrome patients was worse (M = 298.2, SD = 66.5) than that of the general population (M = 360.9, SD = 79.9), with a large Cohen's *d* value of 0.9. The “wellness outlook” of the depressed stagnation syndrome patients appeared grimmer (M = 252.3, SD = 52.2). The correlation between stagnation and depression was higher for affective symptoms than somatic symptoms. Physical distress did not mediate the relationship between stagnation and daily functioning. These might suggest that stagnation syndrome and major depression may share some similar psychological mechanisms.

## 1. Introduction

Stagnation syndrome, a long-established diagnostic entity in traditional Chinese medicine (TCM) classified under internal medicine, is characterized by a number of somatic symptoms without identifiable organic correlates [1]. Since stagnation syndrome also bears close resemblance to the clinical presentations of depression, particularly the somatic form of depression, it has been long regarded as the TCM counterpart of major depression in Western medicine in both clinical practice and research [2]; for example, common symptoms of stagnation syndrome, such as sleeping problems, fatigue, headache, gastrointestinal problems, and emotional restlessness, are also classic symptoms of major depression [3].

Not only the clinical symptoms but also the critical cognitive processes or abnormalities of major depression are observed in stagnation syndrome. Emotion inhibition and rumination are hallmarks of depression and play important roles in the etiology and maintenance of major depression [4–6]. Habitual suppression of emotional experiences and ruminating over problems predict the onset and the severity of major depression [7, 8]. Likewise, as described in TCM classics, the onset of stagnation syndrome is induced by the inhibition of emotions, leading to ‘qi' [vital energy] stagnation that can elicit clinical symptoms when prolonged [9]. The connotation of rumination also resembles a crucial characteristic in stagnation syndrome, namely, overattachment, which indicates the proclivity to be preoccupied with emotional upset about past or future events. Furthermore, the association between stagnation symptoms and depression symptoms has been corroborated by recent empirical research. In an epidemiological study, stagnation symptoms demonstrated a significant positive correlation with depression symptoms (r = .59) [2]. This association is not unique to Chinese people. In studying a consecutive sample of 310 patients with medication-overuse headache, Inamorati (2015) reported a slightly stronger positive association between stagnation symptoms and depression symptoms (r = 0.67) [10].

While the evidence seems to imply that stagnation syndrome and major depression are closely associated and possibly overlap to a great extent, the argument is not conclusive. There is also evidence supporting the view that stagnation and depression are distinct syndromes distinguishable in terms of conceptualization, clinical presentations, and patients' profile [1]. However, current studies touching on the relationship between stagnation syndrome and major depression are only correlational, and the extent to which stagnation syndrome overlaps and co-occurs with major depression has yet to be empirically investigated.

A relatively safer assertion may be that stagnation syndrome can be comprehended as a type of somatic symptom disorder composed of multiple medically unexplained symptoms or functional somatic symptoms that are psychosomatic in essence. Extensive research has reported the high co-occurrence of somatic symptom disorder and major depression [11–13]. Among somatizing patients, elevated functional impairments and abnormal illness behaviors lead to disproportionately high medical costs [14]. However, in clinical practice, the current diagnosis and classification of somatic symptom disorder are inconsistent and often met with confusion [15]. The study of stagnation syndrome, an illness entity with long-established assessment and treatment methods in TCM, may help to shed new light on our understanding and intervention strategies not only for major depression but also for somatic symptom disorder. The study of stagnation syndrome in relation to major depression can also be of useful clinical value considering the relatively high prevalence rate of stagnation syndrome (6.2%) compared with major depression (1.7%) that has previously been reported in Hong Kong and its nonpsychiatric connotation [2, 16]. Stagnation syndrome can be a more embracing concept to use in engaging and working with people in distress.

Therefore, the main aim of this study was to investigate the overlap between major depression and stagnation syndrome. Specifically, the objectives were (a) to estimate the point prevalence of major depression among TCM outpatients suffering from stagnation syndrome, (b) to examine the association between depression and stagnation symptoms, and (c) to evaluate the impacts on wellness of stagnation patients caused by major depression. Since stagnation syndrome is characterized by heightened somatic symptoms, we were also interested in exploring the role of physical distress in impacting functional outcomes.

## 2. Methods

### 2.1. Study Design

This cross-sectional study was based on the data collected for a randomized controlled trial on stagnation syndrome. The analysis of the present study utilized the data from the initial baseline assessments, when no additional treatment apart from the usual herbal and/or acupuncture treatments provided by the TCM clinic had be given. Ethical approval was obtained from the Institutional Review Board of The University of Hong Kong and the West Hong Kong Island Cluster, Hong Kong Hospital Authority.

### 2.2. Participants

Participants were recruited from two TCM clinics operated by nongovernmental medical institutions in Hong Kong. The inclusion criteria were as follows: (a) a score of 50 or above on the Stagnation Scale [1]; (b) diagnosed with stagnation syndrome by a TCM practitioner according to TCM criteria; (c) aged 18 to 60; and (d) no evidence of concurrent life-threatening medical conditions, such as cancer.

The recruitment adopted the 100% consecutive sampling scheme and involved two steps. First, a trained research assistant was stationed in the TCM clinics during the predesignated recruitment periods. An initial assessment utilizing the Stagnation Scale and a screening interview was conducted on all of the patients who gave written consent to participate in the study. Second, potential eligible patients [meeting criteria a, c, and d] were invited for a more thorough diagnostic interview with a licensed TCM practitioner and expert. After the first-stage screening, 162 patients were scheduled for diagnostic interview. Subsequently, 135 patients attended the interview and met all the inclusion criteria of the study. Among these 135 eligible patients, 117 (86%) consented to participate in the study.

### 2.3. Measures

#### 2.3.1. Stagnation Scale

The Stagnation Scale is a 16-item scale measuring the presence and severity of stagnation syndrome [1, 2]. It consists of three subscales: body-mind obstruction, affect-posture inhibition, and overattachment. It has a theoretical range of 16 to 160, with a high score indicating a higher level of stagnation symptoms. The scale has good internal consistency, with Cronbach's alpha coefficients of .91 for the whole scale and between .82 and .88 for the subscales. A cutoff of a total score of 50 was suggested for screening for stagnation syndrome and was adopted in this study for screening, with false-positive and false-negative rates estimated at 26% and 23%, respectively.

#### 2.3.2. Patient Health Questionnaire-9 Chinese Version (PHQ-9)

The PHQ-9 is a screening instrument to facilitate the identification of major depression in primary care settings [17]. It consists of nine items in total, each item corresponding to one of the nine DSM-V criteria of major depression. In accordance with DSM-V, each item is rated on the basis of the patient's report of the extent to which each symptom has persisted in the past 2 weeks: 0 (not at all), 1 (several days), 2 (more than half the days), or 3 (nearly every day) (Table 1). Major depression is diagnosed if the following two requirements are met: (a) five or more of the nine symptoms have lasted for more than half of the days in the past 2 weeks; (b) at least one of the symptoms is depressed mood or anhedonia. The item on thoughts of death was counted if it was present at all [18]. The PHQ-9 indicates the severity of depression symptoms by counting the sum score: 1–4 indicates minimal depression, 5–9 mild depression, 10–14 moderate depression, 15–19 moderately severe depression, and ≥20 severe depression [19]. In previous studies, the Cronbach's alpha of the Chinese version of the PHQ-9 has ranged from 0.79 to 0.89 [15, 16, 20]. The Cronbach's alpha coefficient in the present study was .82. A score of 10 is recommended as the cutoff for identifying major depression [21]. In a current meta-analysis, over 36 studies indicated that the pooled sensitivity and pooled specificity for the cutoff point 10 were 0.78 and 0.87, respectively [22]. For Chinese adults, a cutoff point of 10 was revealed to have a sensitivity of 0.86 and a specificity of 0.94 [20].

#### 2.3.3. Body-Mind-Spirit Well-Being Inventory [BMSWI]

The BMSWI is a multidimensional instrument measuring the level of holistic well-being, including both physical and mental wellness. It comprises four subscales: A, physical distress; B, daily functioning; C, affect; and D, spirituality [23]. The scale demonstrated satisfactory internal consistency in the current study, with a Cronbach's alpha coefficient of .93 for the entire inventory.

The BMSWBI-A is a measure assessing the level of subjective distress caused by multiple somatic symptoms experienced in the past week. The scale consists of 14 items on somatic symptoms; the possible scores range from 0 to 140, with a high score indicating a high level of physical distress. The scale exhibited satisfactory internal consistency in the present sample (Cronbach's alpha coefficient of .88).

The BMSWBI-B measures a patient's evaluation of their everyday functioning in the past week. The scale consists of 10 items that cover a respondent's energy level, levels of concentration, and levels of work motivation. The score range is from 0 to 100, with a high score indicating a high level of functioning. The scale exhibited satisfactory internal consistency in the present sample (Cronbach's alpha coefficient of .85).

The BMSWBI-C subscale measures an individual's levels of positive and negative affect. The scale comprises eight items on positive affect and 11 items on negative affect. The possible scores range from 0 to 80 and from 0 to 110, respectively; a high score denotes a high level of the affect concerned. The scale exhibited satisfactory internal consistency in the present sample (Cronbach's alpha coefficient of .90).

The BMSWBI-D measures the level of an individual's spiritual wellness, including tranquility, resistance to disorientation, and resilience. Spirituality underpins the wisdom to accept unchangeable circumstances and to see difficulties as challenges. The scale consists of 13 items asking the respondent to rate their state of mental tranquility, attitude toward hardship, motivation of life, and so forth on a scale from 0 to 10. Higher scores indicate a higher level of spiritual wellness. The scale had satisfactory internal consistency in the present sample (Cronbach's alpha coefficient of .85).

### 2.4. Statistical Analysis

Descriptive data were presented as means and standard deviations (SD) for the continuous variables and as absolute and relative frequencies for the categorical variables. To test the effect of demographic characteristics on the PHQ-9 and stagnation, independent samples* t*-tests were conducted for the continuous variables and *χ* 2 analyses were conducted for the categorical variables. Pearson correlation analyses were used to test the association between PHQ-9 total and each PHQ-9 item with the stagnation total and its three subscales. The effect sizes for the between-group difference on BMSWI and each subscale were calculated with Cohen's* d*, with values of 0.2, 0.5, and 0.8 suggesting small, medium, and large effect sizes, respectively. SPSS version 21.0 was utilized for the analysis. In addition, mediation analysis was conducted using the R lavaan package. A two-sided *α* level of 0.05 was considered as statistically significant.

## 3. Results

### 3.1. Sample Characteristics

The mean age of the patients in the current study was 48.5 years (SD = 8.7). The median age was 51.0, higher than that of the general population in Hong Kong (median = 43.5) [24]. Eighty-one percent of the patients were female. Over half of the participants were married/cohabiting (61.9%), 24.6% were single, and 13.5% were divorced/separated or widowed. The labor force participation rate (including part-time and full-time) was 66.9%, as compared with 60.3% for the Hong Kong general population.

The education levels of the patients in the current sample were also higher than those of the Hong Kong general population. Around 51% of the patients had received postsecondary education, while the corresponding figure for the Hong Kong general population aged 15 and over was 33.2% in 2016. Among the sample, 22.0% had less than a high school education and 26.5% had completed high school. No significant effects of demographic characteristics on stagnation and depression were found.

The average Stagnation Scale score was 82.1 (SD = 24.9), suggesting a moderately severe level of stagnation syndrome. The mean PHQ-9 score was 10.6 (SD = 4.7). Table 1 summarizes the demographic characteristics and the mean scores for the Stagnation Scale and PHQ-9.

### 3.2. Major Depression Prevalence Rate and Severity of Depression Symptoms

Thirty-one (26.5%) patients with stagnation syndrome met the DSM–V diagnostic criteria of major depression. The mean PHQ-9 score of the depressed patient group was 16.8 (SD = 2.6), whereas the nondepressed patient group had an overall mean score of 8.4 (SD = 2.9), with a Cohen's* d *value of 3.1. The mean stagnation score of the depressed patients was 101.4 (SD = 21.4), and the mean for the nondepressed patients was 75.2 (SD = 22.5), with a Cohen's* d *value of 1.2.

To further examine the severity of depression symptoms, the patients were divided into six categories according to their PHQ-9 scores. All the patients had at least one of the nine depression symptoms. The proportion of patients whose PHQ-9 total score met the cutoff score of 10 was 53%, indicating a moderate and above severity level of depression symptoms.

There was an association between the severity level of depression symptoms and stagnation score [F (4,113(=14.5, p < .001]. This suggests that the more severe the depression symptoms a stagnation syndrome patient experiences, the more distressing the stagnation syndrome they suffer.  Table 2 shows the detailed proportions of patients with differential depression levels over the total sample and the mean stagnation score for each group.

### 3.3. Profiles of Depression Symptoms among the Total Stagnation Syndrome Patient Sample

**Figure 1:**
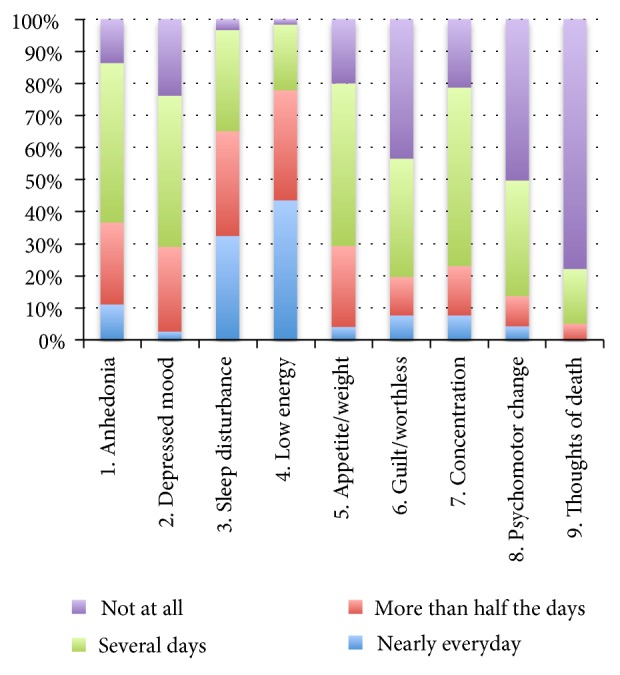
Reports of each depression symptom by percentage for all stagnation syndrome patients (n = 117).

Figure 1 shows the depression symptoms by percentage for all the patients with stagnation syndrome. The depression symptoms that these patients reported as being present for more than half of the days in the previous 2 weeks were somatic symptoms, including low energy (77.8%) and sleep disturbance (65.0%). For the other somatic symptoms, the percentages were relatively lower: 30.8% for appetite/weight change, 23.1% for concentration problems, and 13.7% for psychomotor change. Around one-third of the patients reported experiencing anhedonia (36.7%) and depressed mood (29.1%) for more than half the days, whereas around one-fifth (19.7%) reported a feeling of guilt/worthlessness. In addition, one-fifth of the entire sample reported the presence of thoughts of death (22.2%).

### 3.4. Correlation of Depression Symptoms with Stagnation and Its Subscales

PHQ-9 total score was positively associated with stagnation total score (r = 0.58, p ≤ .01). The three stagnation subscales, body-mind obstruction, affect-posture inhibition, and overattachment, were also positively associated with PHQ-9 total score (r = 0.49, 0.44, 0.49 respectively, all p ≤ .01). However, the strength of the correlation with PHQ-9 was greater for the stagnation total than for each stagnation subscale.

We also computed the correlation of each PHQ-9 item with stagnation total score and the three subscales scores. All the PHQ-9 items were found to be positively correlated with stagnation total score. Among the nine PHQ-9 items, depressed mood (item 2) and guilt/worthlessness (item 6) had the largest correlations with stagnation total score (r = 0.51, 0.48, respectively, all p ≤ .01), whereas sleep disturbance (item 2) and thoughts of death (item 9) had the smallest correlations with stagnation total score (r = 0.24 for both, p ≤ 0.05). Five of the PHQ-9 items (items 1, 3, 5, 6, 9) had a correlation with stagnation total score greater than the correlation with each of the stagnation subscales.  Table 3 depicts the correlations of stagnation total score with PHQ-9 total score and each PHQ-9 item, respectively.

### 3.5. Holistic Wellness of the Depressed Stagnation Syndrome Patients and the Total Stagnation Syndrome Patient Sample in Comparison to General Population

We utilized the data for the general population as the benchmark to compare the holistic wellness of the general population with that of the total stagnation syndrome patient sample and the depressed stagnation syndrome patients, respectively.  Table 4 depicts the BMSWI mean scores of the depressed stagnation syndrome patients, nondepressed stagnation syndrome patients, the total stagnation syndrome patient sample, and the general population and the effect sizes of their between-group differences.

Overall, in comparison to the holistic wellness of the general population (M = 360.9, SD = 79.9), the well-being of the stagnation syndrome patients was much worse (M = 298.2, SD = 66.5). The Cohen's* d *for the between-group difference was 0.9, suggesting a large effect size. Large effect sizes were also found in the mean difference between the total stagnation syndrome patient sample and the general population on two BMSWI subscales, namely, physical distress and daily functioning. The stagnation syndrome patients (M = 73.2, SD = 27.9) suffered from a higher level of physical distress than the general population (M = 29.7, SD = 22.0), with a Cohen's* d *value of 1.1, the greatest among all the BMSWI subscales. The stagnation syndrome patients (M = 36.2, SD = 9.7) also suffered from a more weakened daily functioning than the general population (M = 59.2, SD =16.1), with a Cohen's* d* value of -0.8. For the other two subscales, affect and spirituality, the group difference between the stagnation syndrome patients and the general population showed a medium level of effect size, with a Cohen's* d* value of -0.5 and 0.6, respectively.

With regard to the depressed stagnation syndrome patients (M = 252.3, SD = 52.2), there was a greater contrast between their holistic wellness and that of the general population (M = 360.9, SD = 79.9), and their holistic wellness was significantly jeopardized, as indicated by the high Cohen's* d* value of -1.6 that greatly exceeded the conventional value for large effect size [0.8]. The mean differences with the general population in four BMSWI subscales all showed a large effect size. Similarly, for the total stagnation syndrome patient sample, the aspects of wellness that were most impaired were physical distress and daily functioning, as indicated by an absolute Cohen's d value of 1.7 for both aspects. In addition, affect (Cohen's* d* = -1.2), particularly negative affect (Cohen's* d* = 1.2), and spirituality (Cohen's* d* = -1.1), within which disorientation showed the largest effect size (Cohen's* d* = 1.2), showed a significant difference from the general population.

### 3.6. Mediation Analysis

Mediation analysis was conducted to identify the process underlying the debilitating daily functioning resulting from stagnation. Specifically, we wanted to know whether, for the stagnation syndrome patients, subjective physical concerns caused the adverse functional consequences.

The results of the regression analysis showed that stagnation was a significant predictor of physical distress (b = 0.62, SE = 0.08, p < .001) and that physical distress significantly predicted daily functioning (b = -0.25, SE = 0.05, p <. 05). However, using the estimation approach with 1,000 samples to test the significance of the indirect effect, the bootstrapped standardized indirect effect was not significant (b = -0.15, SE = 0.09, 95% CI = -0.15, -0.15, P = 0.091), suggesting no mediation effect.

## 4. Discussion

This study is the first report to investigate the overlap between major depression, a bio-medical mental disorder originating in Western medicine, and stagnation syndrome, a body-mind connected syndrome rooted in TCM. The results of our study showed that, among the stagnation syndrome patients, comorbidity with major depression was alarmingly high. More than one-quarter (26.5%) of the stagnation syndrome patients met the DSM-V diagnostic criteria for major depression, a rate much higher than that in the general population. The prevalence rate of major depression previously reported in the Chinese general population ranged from 1.1% to 2.4% [25–27]. Previous research using the same instrument (PHQ-9) reported a point prevalent rate of 1.7% in the Hong Kong general population. In our study, the proportion of stagnation syndrome patients exceeding the PHQ-9 cutoff for major depression was also large: over half (53%) of the patients scored over 10, similar to the rate reported among patients with somatic symptoms disorder in China (59.1%) [15], but in great contrast to the proportion reported among the general population in Hong Kong (7.0%) and mainland China (5.6%) [16, 17].

The impairment in holistic wellness among the stagnation syndrome patients was also pronounced and worthy of attention. Our results revealed that the stagnation syndrome patients experienced considerably worse physical and emotional health than the general population. Heightened subjective physical distress and debilitating daily functioning were the most conspicuous features of their wellness outlook. The comorbidity of stagnation syndrome with major depression could result in even more exacerbated physical and mental problems. This is consistent with the results of previous studies that indicate that the co-occurrence of somatic symptom disorder with major depression would result in worse clinical and functional outcomes and adversely impact on quality of life [28, 29].

In addition to subjective physical distress and daily functioning, the depressed stagnation syndrome patients were also significantly distinguished from the general population by their deficiency in positive affect, excess of negative affect, and feelings of overwhelming disorientation. In contrast, these differences were small between all the stagnation syndrome patients and the general population. This result is consistent with the conclusion in a previous study that stagnation syndrome and depression might differ in their clinical manifestation on affect [2]. While the connotation of depression implies in itself an intrinsic meaning of low mood, negativity, and deprivation of positivity and hopefulness, stagnation does not necessarily suggest that a person's mood has to be downcast; rather, it denotes a mood state that is not flowing, and when a person's mood is not flowing and functioning, it becomes easier to become low/depressed if symptoms escalate. This might also explain why depression symptoms tend to be more severe among patients suffering from more intensive stagnation.

Furthermore, the results of this study strengthen the argument that although stagnation syndrome is defined as an internal illness with predominantly somatic symptoms, it might be in nature a dysfunction of emotion. In the present study, the associations with the affective aspects of depression symptoms [depressed mood & guilt/worthlessness] were greater in strength than the associations with the somatic aspects. This argument can also be supported by the evidence in the study that around one-third of the stagnation syndrome patients positively reported the presence of anhedonia (36.7%) and depressed mood (29.1%), the key affective symptoms of depression, for more than half the days in the previous 2 weeks. These findings are congruent with the TCM conceptualization of stagnation. Described in Chinese medical classics, stagnation syndrome starts from, and is essentially a consequence of, emotion inhibition and imbalance [30]. It is when the emotion inhibition and imbalance are prolonged and become excessive and aggravating that elevated somatic symptoms will be prompted due to a series of disorganized psychophysiological processes being triggered [31, 32]. The stronger association of stagnation syndrome with the affective symptoms of depression than with the somatic symptoms of depression observed in the present study might have significant clinical implications for managing stagnation syndrome patients and draw close comparison with somatic depression, a subtype of depression that has been argued to be different from pure depression [33, 34].

Consistently, the results of present study also disapproved the mediation effect of physical distress between stagnation symptoms and daily functioning. The results demonstrate that patients' daily functioning is not disrupted because of the elevated physical distress caused by stagnation syndrome, despite the fact that stagnation syndrome presents itself primarily in terms of pronounced physical discomfort. A plausible reason for this finding might lie in the impacts of different dimensional aspects of the distress/pain. Previous studies have suggested that the experience of physical distress/pain can be divided into two dimensions: the sensory-discriminative dimension [sensational perception of the pain] and the affective-motivational dimension [negative emotions the pain provokes] [35]. The level of the affective component of the pain can be independent of the sensory component, meaning that one could feel differently with a similar level of pain [36, 37]. It might be the affective dimension of physical distress, not the sensory dimension the present study was concerned with, that engenders the impairment in daily functioning as a result of stagnation. The finding of a previous intervention study for patients with stagnation syndrome might offer some evidence for this postulation. While both the intervention and control groups showed a significant amelioration in subjective physical distress with no between-group difference, it was in the intervention group, where participants had additional exposure to affective/cognitive treatment, that the improvement in social functioning was significantly larger [38]. However, more research is warranted.

Methodologically, the strengths of the present study include a rigorous procedure for recruitment and the diagnosis for stagnation syndrome, the adoption of the 100% consecutive sampling scheme, and a high response rate. However, a number of limitations should be noted. First, our sample was comprised of stagnation syndrome patients who sought treatment at TCM clinics, and thus the findings may not be generalizable to people who suffer from stagnation syndrome in the community and do not resort to any treatment or seek treatment in other settings, such as public hospitals. Self-selection bias might exist due to the choosing of TCM clinics as the study sites for recruitment, as manifested in the differences in demographic profile between the current sample and the general population (e.g., higher education and income level). However, these characteristics of our sample are in line with the findings of a previous epidemiology investigation, where stagnation syndrome was found to be more prevalent among people with a better education and a middle-upper level of income. Second, the sample size in the present study was relatively small. Third, the current study was cross-sectional in design, and thus the causal relationships suggested by the results are only tentative. Future studies focused on a large sample size and with a longitudinal design are much needed.

## Figures and Tables

**Table 1:** Demographic characteristics and the mean scores for Stagnation Scale and PHQ-9.

Demographic Variables	N	Percentage	Mean ± SD	
			PHQ-9	Stagnation
**Age **(Mean ± SD)	117	48.5 ± 8.7	10.6 ± 4.7	82.1 ± 24.9
**Gender**				
Female	95	81.4	10.5 ± 4.5	81.2 ± 24.6
Male	22	18.6	11.0 ± 5.4	86.2 ± 25.9
**Marital Status**				
Single	29	24.6	10.4 ± 4.4	86.3 ± 24.0
Married/cohabiting	72	61.9	10.3 ± 4.9	80.4 ± 25.3
Divorced/separated	9	7.6	13.2 ± 4.5	84.6 ± 32.2
Widow	7	5.9	10.9 ± 2.6	79.7 ± 13.1
**Employment**				
Full-time	65	55.9	10.1 ± 4.0	84.4 ± 25.0
Part-time	12	11	13.1 ± 4.5	90.3 ± 23.5
Retired	9	7.6	12.8 ± 6.9	78.9 ± 20.6
Homemaker	25	20.3	9.8 ± 5.3	72.7 ± 27.1
Unemployed	6	5.1	11.8 ± 3.3	85.0 ± 14.4
**Education Level**				
Primary school	6	5.1	12.2 ± 3.9	91.5 ± 13.3
Middle school	20	17.1	11.0 ± 5.7	77.2 ± 29.7
High school	31	26.5	11.0 ± 5.1	80.7 ± 29.4
College or above	60	51.3	10.1 ± 4.2	83.4 ± 21.4
**Income (HK$)**				
None	33	28	10.7 ± 5.6	76.8 ± 26.6
<$10,000	14	11.9	13.3 ± 4.5	90.6 ± 23.1
$10,000-$19,999	19	16.1	10.8 ± 4.3	80.0 ± 21.9
$20,000-$39,999	25	21.2	10.1 ± 3.9	79.9 ± 27.2
≥ $40,000	26	22.9	9.4 ± 4.1	87.7 ± 22.4

**Table 2:** Percentage of patients with differential depression levels over total sample and the stagnation mean score in each group (n = 117).

Level of depression symptoms	Definition	Percentage	Stagnation total mean (SD)
Minimal	PHQ-9 = 1-4	7.7%	46.1(16.1)
Mild	PHQ-9 = 5-9	39.3%	75.8(21.3)
Moderate	PHQ-9 = 10-14	30.8%	84.6(19.1)
Moderate severe	PHQ-9 = 15-19	17.1%	100.4(21.0)
Severe	PHQ-9 = 20-27	5.1%	108.8(28.1)

Total		100%	82.1(24. 9)

Major depression	Presence of 5 or more symptoms, and either depressed mood or anhedonia must be included	26.5%	101.4(21.4)

**Table 3:** Correlations of Stagnation Scale total score and three subscales with PHQ-9 total score and each depression symptom (n = 117).

PHQ-9 items	Stagnation total	Stagnation Subscale		
		Body-mind obstruction	Affect-posture inhibition	Over-attachment
(1) Anhedonia	0.43*∗∗*	0.29*∗∗*	0.41*∗∗*	0.37*∗∗*
(2) Depressed mood	0.51*∗∗*	0.46*∗∗*	0.29*∗∗*	0.54*∗∗*
(3) Sleep disturbance	0.24*∗*	0.12	0.22*∗*	0.23*∗*
(4) Low energy	0.33*∗∗*	0.33*∗∗*	0.29*∗∗*	0.21*∗*
(5) Appetite/weight	0.35*∗∗*	0.33*∗∗*	0.20*∗*	0.30*∗∗*
(6) Guilt/worthless	0.48*∗∗*	0.35*∗∗*	0.34*∗∗*	0.45*∗∗*
(7) Concentration	0.39*∗∗*	0.41*∗∗*	0.27*∗∗*	0.29*∗∗*
(8) Psychomotor change	0.36*∗∗*	0.39*∗∗*	0.30*∗∗*	0.22*∗*
(9) Thoughts of death	0.24*∗*	0.23*∗*	0.18*∗*	0.18*∗*
PHQ-9 total	0.58*∗∗*	0.49*∗∗*	0.44*∗∗*	0.49*∗∗*

*∗∗* Correlation is significant at the 0.01 level (2-tailed).

*∗* Correlation is significant at the 0.05 level (2-tailed).

**Table 4:** BMSWI mean scores of depressed stagnation patients, nondepressed stagnation patients, total stagnation patient sample, and general population and the effect sizes of their between-group difference.

BMSWI subscale	Mean (SD)				Cohen's d^1^	
	Stagnation depressed (n = 31)	Stagnation non-depressed (n = 86)	Stagnation total (n = 110)	General population^2^ (n = 816)	Stagnation depressed vs. general population	Stagnation total vs. general population
Physical Distress	73.2(27.9)	51.7(24.4)	57.4(27.0)	29.7(22.0)	1.7	1.1
Daily Functioning	36.2(9.7)	50.8(12.0)	46.9(13.1)	59.2(16.1)	-1.7	-0.8
Affect total	73.3(26.5)	104.6(24.1)	96.2(28.3)	110.7(32.3)	-1.2	-0.5
Positive Affect	28.8(15.7)	38.8(11.4)	36.2(13.4)	42.3(14.9)	0.9	0.4
Negative Affect	65.6(18.7)	44.3(18.0)	50.0(20.4)	40.7(22.1)	1.2	0.4
Spirituality	61.5(22.5)	76.7(17.9)	72.7(20.3)	85.4(22.8)	-1.1	0.6
Tranquility	21.0(11.5)	25.9(8.2)	24.6(9.4)	28.6(10.7)	0.7	0.4
Disorientation	27.7(11.2)	16.4(9.5)	19.4 (11.1)	14.5(11.0)	1.2	0.4
Resilience	17.8(7.2)	17.3(6.3)	17.5(6.6)	21.5(5.9)	-0.6	0.6
BMSWI total	252.3(52.2)	324.6 (59.2)	298.2(66.5)	360.9(79.9)	-1.6	0.9

Note: ^1^Cohen's *d* with values of 0.2, 0.5, and 0.8 suggest small, medium, and large effect sizes respectively; ^2^subjects were Chinese adults in the community of Hong Kong.

## References

[1] Ng S.-M., Fong T. C. T., Wang X.-L., Wang Y.-J. (2012). Confirmatory factor analysis of the stagnation scale-a traditional chinese medicine construct operationalized for mental health practice. *International Journal of Behavioral Medicine*.

[2] Ng S.-M., Chan C. L. W., Ho D. Y. F., Wong Y.-Y., Ho R. T. H. (2006). Stagnation as a distinct clinical syndrome: Comparing 'Yu' (stagnation) in traditional Chinese medicine with depression. *British Journal of Social Work*.

[3] Scheid V. (2013). Depression, Constraint, and the Liver: (Dis)assembling the Treatment of Emotion-Related Disorders in Chinese Medicine. *Culture, Medicine and Psychiatry*.

[4] Barlow D. H., Allen L. B., Choate M. L. (2016). Toward a Unified Treatment for Emotional Disorders – Republished Article. *Behavior Therapy*.

[5] Lee S., Tsang A., Huang Y.-Q. (2009). The epidemiology of depression in metropolitan China. *Psychological Medicine*.

[6] Koster E. H. W., De Lissnyder E., Derakshan N., De Raedt R. (2011). Understanding depressive rumination from a cognitive science perspective: The impaired disengagement hypothesis. *Clinical Psychology Review*.

[7] Campbell-Sills L., Barlow D. H., Brown T. A., Hofmann S. G. (2006). Effects of suppression and acceptance on emotional responses of individuals with anxiety and mood disorders. *Behaviour Research and Therapy*.

[8] Gross J. J., John O. P. (2003). Individual differences in two emotion regulation processes: implications for affect, relationships, and well-being. *Journal of Personality and Social Psychology*.

[9] Zhang Y. H. (2012). *Transforming emotions with Chinese medicine: An ethnographic account from contemporary China*.

[10] Innamorati M., Pompili M., Erbuto D. (2015). Psychometric properties of the stagnation scale in medication overuse headache patients. *The Journal of Headache and Pain*.

[11] Henningsen P., Löwe B. (2006). Depression, pain, and somatoform disorders. *Current Opinion in Psychiatry*.

[12] Löwe B., Spitzer R. L., Williams J. B. W., Mussell M., Schellberg D., Kroenke K. (2008). Depression, anxiety and somatization in primary care: syndrome overlap and functional impairment. *General Hospital Psychiatry*.

[13] Steinbrecher N., Koerber S., Frieser D., Hiller W. (2011). The Prevalence of Medically Unexplained Symptoms in Primary Care. *Psychosomatics*.

[14] Barsky A. J., Orav E. J., Bates D. W. (2005). Somatization increases medical utilization and costs independent of psychiatric and medical comorbidity. *Archives of General Psychiatry*.

[15] Xiong N., Fritzsche K., Wei J. (2015). Validation of patient health questionnaire (PHQ) for major depression in Chinese outpatients with multiple somatic symptoms: A multicenter cross-sectional study. *Journal of Affective Disorders*.

[16] Yu X., Tam W. W. S., Wong P. T. K., Lam T. H., Stewart S. M. (2012). The Patient Health Questionnaire-9 for measuring depressive symptoms among the general population in Hong Kong. *Comprehensive Psychiatry*.

[17] Wang W., Bian Q., Zhao Y. (2014). Reliability and validity of the Chinese version of the Patient Health Questionnaire (PHQ-9) in the general population. *General Hospital Psychiatry*.

[18] American Psychiatric Association (2013). *Diagnostic and statistical manual of mental disorders*.

[19] Kroenke K., Spitzer R. L., Williams J. B. W. (2001). The PHQ-9: validity of a brief depression severity measure. *Journal of General Internal Medicine*.

[20] Liu S.-I., Yeh Z.-T., Huang H.-C. (2011). Validation of Patient Health Questionnaire for depression screening among primary care patients in Taiwan. *Comprehensive Psychiatry*.

[21] Spitzer R. L., Kroenke K., Williams J. B. W. (1999). Validation and utility of a self-report version of PRIME-MD: the PHQ Primary Care Study. *Journal of the American Medical Association*.

[22] Moriarty A. S., Gilbody S., McMillan D., Manea L. (2015). Screening and case finding for major depressive disorder using the Patient Health Questionnaire (PHQ-9): A meta-analysis. *General Hospital Psychiatry*.

[23] Ng S. M., Yau J. K. Y., Chan C. L. W., Chan C. H. Y., Ho D. Y. F. (2005). The measurement of Body-Mind-Spirit well-being: Toward multidimensionality and transcultural applicability. *Social Work in Health Care*.

[24] Government of the Hong Kong Special Administrative Region (2016). Thematic Report: Household income distribution in Hong Kong. *Hong Kong 2016 Population*.

[25] Phillips M. R., Zhang J., Shi Q. (2009). Prevalence, treatment, and associated disability of mental disorders in four provinces in China during 2001-05: an epidemiological survey. *The Lancet*.

[26] Kessler R. C., Birnbaum H. G., Shahly V. (2010). Age differences in the prevalence and co-morbidity of DSM-IV major depressive episodes: results from the WHO world mental health survey initiative. *Depression and Anxiety*.

[27] Liu J., Yan F., Ma X. (2015). Prevalence of major depressive disorder and socio-demographic correlates: Results of a representative household epidemiological survey in Beijing, China. *Journal of Affective Disorders*.

[28] Chakraborty K., Avasthi A., Kumar S., Grover S. (2012). Psychological and clinical correlates of functional somatic complaints in depression. *International Journal of Social Psychiatry*.

[29] Henningsen P., Jakobsen T., Schiltenwolf M., Weiss M. G. (2005). Somatization revisited: Diagnosis and perceived causes of common mental disorders. *The Journal of Nervous and Mental Disease*.

[30] Zhang H.-L. (2010). Treatment of depression based on differentiation of the shaoyang channels. *Journal of Traditional Chinese Medicine*.

[31] Zhao G. X. (2009). *Zhao Qingli’s regulation treatment of constraint patterns with case records and medical essays*.

[32] Qiao M. Q., Zhang M. Y. (2009). *The study of emotions in Chinese medicine*.

[33] Silverstein B. (2002). Gender differences in the prevalence of somatic versus pure depression: A replication. *The American Journal of Psychiatry*.

[34] Wardenaar K. J., Monden R., Conradi H. J., De Jonge P. (2015). Symptom-specific course trajectories and their determinants in primary care patients with Major Depressive Disorder: Evidence for two etiologically distinct prototypes. *Journal of Affective Disorders*.

[35] Harvey A. M. (1995). Classification of Chronic Pain—Descriptions of Chronic Pain Syndromes and Definitions of Pain Terms. *The Clinical Journal of Pain*.

[36] Lerman S. F., Rudich Z., Shahar G. (2010). Distinguishing affective and somatic dimensions of pain and depression: A confirmatory factor analytic study. *Journal of Clinical Psychology*.

[37] Maihöfner C., Herzner B., Otto Handwerker H. (2007). Secondary somatosensory cortex is important for the sensory-discriminative dimension of pain: A functional MRI study. *European Journal of Neuroscience*.

[38] Ng S. M., Wang Q. Body-mind-spirit group therapy for Chinese medicine stagnation syndrome - RCT with self-report and physiological measures.

